# Checkpoint inhibitors as immunotherapy for fungal infections: Promises, challenges, and unanswered questions

**DOI:** 10.3389/fimmu.2022.1018202

**Published:** 2022-10-25

**Authors:** Sebastian Wurster, Stephanie S. Watowich, Dimitrios P. Kontoyiannis

**Affiliations:** ^1^ Department of Infectious Diseases, Infection Control and Employee Health, The University of Texas MD Anderson Cancer Center, Houston, TX, United States; ^2^ Department of Immunology, The University of Texas MD Anderson Cancer Center, Houston, TX, United States

**Keywords:** immunotherapy, fungal sepsis, candidiasis, aspergillosis, mucormycosis, checkpoint inhibitors, T cells, immune exhaustion

## Abstract

Opportunistic fungal infections have high mortality in patients with severe immune dysfunction. Growing evidence suggests that the immune environment of invasive fungal infections and cancers share common features of immune cell exhaustion through activation of immune checkpoint pathways. This observation gave rise to several preclinical studies and clinical case reports describing blockade of the Programmed Cell Death Protein 1 and Cytotoxic T-Lymphocyte Antigen 4 immune checkpoint pathways as an adjunct immune enhancement strategy to treat opportunistic fungal infections. The first part of this review summarizes the emerging evidence for contributions of checkpoint pathways to the immunopathology of fungal sepsis, opportunistic mold infections, and dimorphic fungal infections. We then review the potential merits of immune checkpoint inhibitors (ICIs) as an antifungal immunotherapy, including the incomplete knowledge of the mechanisms involved in both immuno-protective effects and toxicities. In the second part of this review, we discuss the limitations of the current evidence and the many unknowns about ICIs as an antifungal immune enhancement strategy. Based on these gaps of knowledge and lessons learned from cancer immunology studies, we outline a research agenda to determine a “sweet spot” for ICIs in medical mycology. We specifically discuss the importance of more nuanced animal models, the need to study ICI-based combination therapy, potential ICI resistance, the role of the immune microenvironment, and the impact of ICIs given as part of oncological therapies on the natural immunity to various pathogenic fungi.

## Introduction

Conventional antifungal therapy for the treatment of invasive fungal infections (IFIs) remains challenging due to a limited number of available drugs, considerable toxicities, drug-drug interactions, and the increasing global spread of resistant pathogens such as azole-resistant *Aspergillus fumigatus* and echinocandin-resistant *Candida* species (1, 2). Investigational antifungal agents currently studied in phase 2 or 3 trials continue to have major gaps in their therapeutic spectrum, especially limited activity against Mucorales and *Fusarium* species (3). Furthermore, despite improved clinical management and the introduction of new antifungal agents, outcomes of IFIs remain dismal in patients with profound immune dysfunction, e.g., patients with persistent neutropenia or refractory leukemia (4, 5). Therefore, the development of facile immune enhancement strategies to potentiate the efficacy of conventional antifungals remains an important pursuit (6).

Over the past 20 years, many groups designed cellular immune therapeutics to restore and augment antifungal immunity, including adoptive T cell transfer, chimeric antigen receptor T cells, *ex-vivo*-pulsed dendritic cells (DCs), and azole-loaded neutrophils (7–12). However, despite a multitude of promising preclinical data, only few of these approaches eventually entered small-scale clinical studies, which is not surprising, as cellular immune therapeutics are costly, difficult to scale, time- and labor-intensive, logistically challenging, and subject to considerable regulatory hurdles (11, 12). These obstacles for clinical translation of cellular immune therapeutics have reinvigorated the interest in non-cellular immune enhancement strategies to bolster host defense against opportunistic pathogens (12). For instance, recombinant interferon-gamma (IFN-γ), a cytokine that is considered a pivotal driver of protective antifungal immunity, has been studied as an adjunct immune-stimulatory therapy in patients with IFIs (13). Furthermore, recombinant hematopoietic growth factors (GM-CSF and G-CSF) were shown to improve fungicidal immunity in mouse models (14, 15), are commonly used in the management of congenital and acquired neutropenia (16), and have been studied as an adjunct immunotherapeutic approach in patients with IFIs (17, 18).

Increasing evidence pointing to similarities in the immune pathogenesis of cancers and infections has led to the exploration of existing oncological immune therapeutics, such as immune checkpoint inhibitors (ICIs), as adjunct treatments in infectious diseases (19, 20). As extensively reviewed elsewhere, ICIs were studied in case series or early-stage clinical trials in patients with HIV infection, hepatitis C, progressive multifocal leukoencephalopathy, and bacterial sepsis (19). Furthermore, dozens of preclinical studies have suggested that ICIs could become a facile and effective adjunct immunotherapeutic approach in a broad spectrum of infectious diseases including IFIs (19).

The first part of this review (section 2) examines the evidence from *in-vitro* studies, animal research, and clinical case reports supporting a role of checkpoint pathways as potential therapeutic targets in fungal sepsis, opportunistic mold infections, and dimorphic fungal infections. In the second part (section 3), we discuss the many unknowns about antifungal ICI therapy and outline a research agenda to better understand the “sweet spot” for ICIs in medical mycology based on lessons learned from immuno-oncology studies.

## The growing promise of ICIs in medical mycology

### The role of immune exhaustion and checkpoint pathways in antifungal immunity

**Figure 1:**
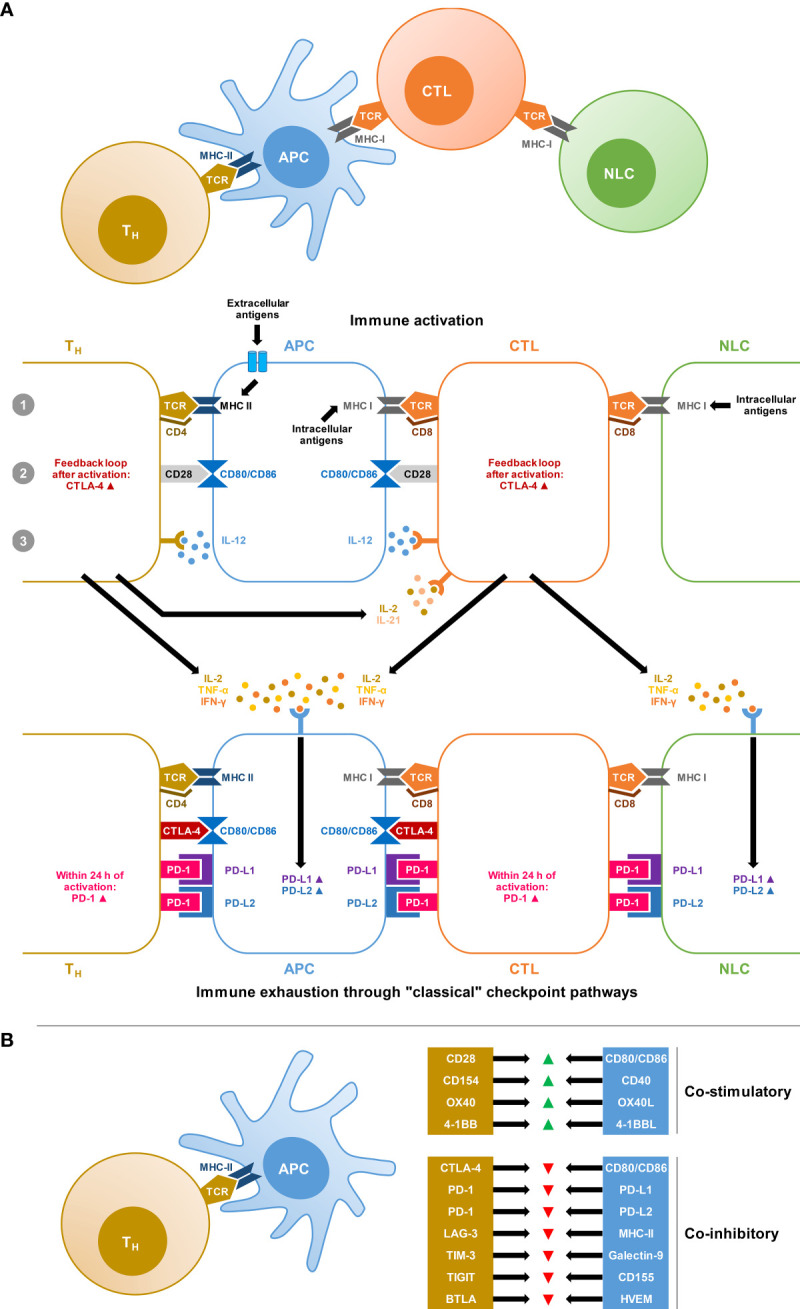
Co-stimulatory and co-inhibitory signals involved in T-cell activation and immune exhaustion. **(A)** Simplified schematic of the “3-signal concept” of T-cell activation by professional antigen-presenting cells (APCs) and non-lymphatic cells (NLCs), i.e., infected tissue or tumor cells. Signal 1: Antigen-loaded Major Histocompatibility Complex (MHC) is recognized by T cells with a corresponding T-cell receptor (TCR) and the interaction is stabilized by CD4 expressed on T-helper (Th) cells or CD8 expressed on cytotoxic T lymphocytes (CTLs). Signal 2: T-cell-expressed CD28 interacts with the B7 complex (CD80 and CD86) on APCs. Signal 3: Additional cytokine signals, especially interleukin-12 (IL-12) produced by APCs are required for full stimulation. This system is equipped with negative feedback loops, resulting in the upregulation of Cytotoxic T-Lymphocyte Antigen 4 (CTLA-4) on T cells, which outcompetes the interaction of CD28 with the B7 complex. Furthermore, chronic antigen exposure and cytokine signals, especially interferon gamma (IFN-γ) stimulate the expression of the Programmed Death Ligands PD-L1 and PD-L2 on APCs and NLCs. These ligands interact with Programmed Cell Death Protein 1 (PD-1), which is upregulated within 24 hours of T-cell activation and initiates inhibitory signaling cascades after ligand binding. In reality, this system is highly complex and further tine-tuned by additional co-stimulatory and co-inhibitory signals and the immune environment. **(B)** Selection of co-stimulatory and co-inhibitory signals in the T-cell/APC interplay undergoing preclinical and/or clinical evaluation as potential targets for oncological immunotherapies. Abbreviations: BTLA, B and T lymphocyte Attenuator; CD, cluster of differentiation; HVEM, Herpes Virus Entry Mediator; LAG-3, Lymphocyte-Activation Gene 3; TIGIT, T Cell Immunoreceptor With Immunoglobulin and ITIM Domain; TIM-3, T cell Immunoglobulin and Mucin Domain-Containing Protein 3.

Activation of T cells by antigen-presenting cells (APCs) and non-lymphoid cells (i.e., infected tissues and tumor cells) is tightly controlled by a balance of co-stimulatory and co-inhibitory signals (**Figure 1A**). Immune checkpoint pathways, that is, co-inhibitory pathways expressed as feedback loops after immune activation or in response to chronic antigen exposure and inflammation, are crucial to induce tolerance to autoantigens and thereby limit autoimmunity (21). However, checkpoint pathways also promote immune exhaustion in scenarios of excessive or chronic antigen exposure due to inflammation, infection, or cancer (21). Although the exact definition of immune exhaustion is debated, the term commonly refers to a hypofunctional state of immune cells, especially T cells, characterized by reduced cytokine production, limited proliferation, epigenetic and metabolic changes, and upregulation of inhibitory receptors (22).

The two major classical checkpoint pathways associated with immune exhaustion are the Cytotoxic T-Lymphocyte Antigen 4 (CTLA-4) pathway and the Programmed Cell Death Protein 1 (PD-1) pathway (21). The CTLA-4 pathway is predominantly involved in the activation of naïve T-cells at the priming stage (21, 23). The most potent co-stimulatory signal for naïve T-cell activation is the interaction of the T-cellular CD28 surface protein with CD80 and CD86 on APCs. To prevent overzealous activation and autoreactivity, the CD28 pathway is equipped with a negative feedback loop through the expression and exocytosis of CTLA-4, which then outcompetes the interaction of CD28 with CD80/CD86 due to its greater affinity, resulting in inhibitory signals (21, 23) (**Figure 1A**).

PD-1 is more broadly expressed on T cells, B cells, natural killer (NK) cells, and mononuclear phagocytes, and is rapidly activated following T-cell receptor (TCR) stimulation (21, 24). PD-1 signaling inhibits T-cell survival and proliferation, suppresses the release of T-cell effector cytokines, and interferes with TCR signaling (21). PD-1 interacts with two main ligands, PD-L1 and PD-L2, that are widely expressed on professional APCs as well as nonlymphoid cells and tissues (**Figure 1A**). IFN-γ, secreted by activated T cells, is considered the most potent stimulus of PD-L1 upregulation (21, 24).

Besides these classical immune checkpoints, additional molecules with co-inhibitory functions in the interplay between T cells and innate immune cells or non-lymphoid tissues are increasingly studied (25) (**Figure 1B**). The best-characterized emerging checkpoint molecules are Lymphocyte-Activation Gene 3 (LAG-3), T cell Immunoglobulin and Mucin Domain-Containing Protein 3 (TIM-3), and T Cell Immunoreceptor With Immunoglobulin and ITIM Domain (TIGIT) (25).

Several studies documented the activation of these immune checkpoint pathways during invasive yeast infections (19). After initial hyperinflammation, sepsis patients often experience a profound anti-inflammatory response with functional impairment of lymphocytes and phagocytes (26–30). For instance, circulating T cells and NK cells from patients and mice with *Candida* sepsis displayed increased expression of immunosuppressive signals, especially PD-1 and PD-L1, and concomitant downregulation of co-stimulatory molecules (31, 32). This sustained immunoinhibitory response contributes to poor outcomes despite appropriate antifungal therapy, including increased risk of secondary infections, and long-lasting immune impairment in survivors (28–30). A recent study expanded these findings by showing strong co-induction of PD-1, LAG-3, and TIM-3 on peripheral blood mononuclear cells (PBMCs) from patients with invasive *C. albicans* infection (29). Notably, the immune environment in patients with invasive candidiasis (IC) partially resembled patterns of immune dysfunction and exhaustion seen in tumor-infiltrating lymphocytes (29). Among the upregulated checkpoint markers, PD-1 showed the strongest association with poor mortality outcomes in IC patients (29). *In-vitro* blockade of PD-1 on patient cells enhanced *C. albicans*-induced Th-cell effector responses, especially the production of the type 1 T-helper cell (Th1) signature cytokine IFN-γ (29). Single-cell RNA sequencing after stimulation of PBMCs from healthy donors with *C. albicans* antigens further corroborated the co-induction of the PD-1 axis, CTLA-4, LAG-3, and other co-inhibitory molecules on Th cells, cytotoxic T lymphocytes (CTLs), and mononuclear phagocytes (33).

Checkpoint activation in non-*albicans Candida* sepsis was demonstrated by Spec and colleagues who assayed co-stimulatory and inhibitory signals on circulating T cells from patients with bloodstream infection (BSI) due to various *Candida* species (28). Compared with non-septic critically ill patients, Th cells and CTLs from patients with *Candida* BSIs showed significantly upregulated PD-1 expression, whereas CD28 was suppressed (28). Furthermore, our recent pilot study of checkpoint induction in an immunocompetent mouse model of *C. auris* sepsis revealed elevated expression of PD-1 on T cells and PD-L1 on macrophages from infected mice (34). PD-L1 expression on macrophages strongly correlated with *C. auris* yeast cell burden in kidney tissue, suggesting that PD-1/PD-L1 checkpoint induction might hamper fungal clearance (34). Similarly, persistent cryptococcal lung infection induced robust and sustained upregulation of PD-1 on Th cells and its ligands PD-L1 and PD-L2 on DCs and alveolar macrophages in a murine infection model (35).

Further, two studies demonstrated profound and sustained immune exhaustion after *Pneumocystis jirovecii* pneumonia (PJP). T and B cells from non-HIV patients with PJP displayed strongly elevated transcription and surface expression of PD-1 and PD-L1 on T and B cells (36). The authors confirmed these findings in a murine PJP model and showed that upregulation of PD-1 on Th cells and CTLs persisted for up to 6 weeks after *Pneumocystis* infection (36). Others found increased PD-1 expression on alveolar macrophages from mice with PJP and pulmonary accumulation of Myeloid-Derived Suppressor Cells (MDSCs) that strongly expressed PD-L1 (37). Furthermore, alveolar macrophages from uninfected mice showed increased PD-1 expression and weaker phagocytic capacity after co-culture with MDSCs from mice with PJP (37). This effect was partially reversible by pretreatment of *ex-vivo* MDSCs with anti-PD-L1 (37).

*In vitro* data and animal studies also support a role of checkpoint upregulation after mold infection or sensitization to mold antigens. Balb/c mice sensitized *via* intraperitoneal and intranasal administration of *Aspergillus fumigatus* allergens showed elevated expression of CTLA-4 with concomitant downregulation of CD28 on pulmonary Th cells (38). This mechanism is thought to protect the host against tissue damage during persistent anti-*Aspergillus* responses, e.g., through regulation of the balance between regulatory T cells (Treg) and proinflammatory type 17 T-helper (Th17) cells or reversal of the proinflammatory phenotype of Treg cells (39, 40). Likewise, others found that *A. fumigatus* cell wall α-(1,3)-glucan stimulated the expression of PD-L1 on human DCs, which in turn promoted polarization of naïve T cells toward Treg cells while suppressing Th1 responses (41). Antibody-mediated blockade of PD-L1 *in vitro* led to significant inhibition of α- (1, 3)-glucan-induced Treg cells while enhancing IFN-γ production by Th1 cells. These findings suggested that PD-L1 on DCs orchestrates the balance between Treg and Th1 responses to *Aspergillus* antigens (41).

Two studies provided insights into the activation of checkpoint pathways during dimorphic fungal infections (42, 43). T-cells from patients with active paracoccidioidomycosis showed increased expression of CTLA-4 and decreased proliferative capacity after stimulation with the strong T-cell activator phytohemagglutinin. Antibody-mediated blockade of CTLA-4 enhanced IFN-γ responses of *ex-vivo* PBMCs from patients with paracoccidioidomycosis (42). Mice infected with *Histoplasma capsulatum* showed broadly upregulated PD-L1 expression on multiple splenic immune cell subsets (43). The suppressive phenotype of macrophages after infection with *H. capsulatum* strongly inhibited proliferation and cytokine secretion by activated T-cells during *in-vitro* co-culture (43).

Collectively, these studies provide solid evidence that co-inhibitory checkpoint pathways are induced by a broad spectrum of pathogenic fungi, such as yeasts, molds, and dimorphic fungi. Moreover, several of these studies revealed a link between upregulated checkpoint pathway expression and signs of impaired antifungal immunity, pointing to a role of checkpoint pathways as therapeutic targets in medical mycology.

### Preclinical studies of ICI therapy in animal models of fungal infections

In 2000, McGaha & Murphy published the first comprehensive *in-vivo* study demonstrating a therapeutic potential of ICIs in experimental mycology. The authors showed that antibody-mediated blockade of CTLA-4 prolonged survival of mice intravenously infected with the highly virulent *C. neoformans* strain NU-2 (44). Additionally, CTLA-4 blockade augmented cell-mediated responses to immunization with the cryptococcal antigen CneF and enhanced immunization-induced protection from subsequent *C. neoformans* infection (44). In another study, treatment with a PD-1 antibody (anti-PD-1) promoted fungal clearance from lungs of mice with persistent *C. neoformans* infection and reduced fungal dissemination to brain and spleen tissue (35). However, in both cryptococcal infection models, attainment of favorable outcomes required high-dose ICI treatment given twice weekly over a course of several weeks (35, 44).

In a sub-lethal PJP mouse model, anti-PD-1 treatment every 3 days for 3 weeks resulted in faster weight gain of both immunocompetent and corticosteroid-immunosuppressed *Pneumocystis*-infected mice (36). However, improved fungal clearance after anti-PD-1 treatment was only seen in immunocompetent mice (36).

Several studies suggested a therapeutic benefit of ICIs alone or in combination with antifungals in mice with primary or secondary *Candida* sepsis. Spec and colleagues compared blockade of PD-1, PD-L1, and CTLA-4 inhibition in both single-hit *C. albicans* sepsis and secondary candidiasis after peritonitis induced by cecal ligation and puncture (CLP) (32). Although the PD-1 pathway inhibitors tended to provide a stronger early survival benefit than anti-CTLA-4 in mice with single-hit candidiasis, improvement in 12-day mortality was comparable between the ICI treatments (32). Similarly, all three inhibitors yielded comparable survival benefits in mice with secondary *C. albicans* sepsis compared with the corresponding isotype controls (32). Notably, all experiments in this study were conducted with concomitant fluconazole therapy in order to document the efficacy of immunotherapy when added to routine antifungal therapy. Others additionally confirmed a mono-therapeutic benefit of CTLA-4 blockade in mice with single-hit *C. albicans* sepsis or secondary candidiasis after CLP (45). Interestingly, an inverse relationship between the anti-CTLA-4 dose and survival was noted. High doses of anti-CTLA-4, as used in preclinical cancer immunotherapy studies (“oncological dosing”), worsened survival, whereas a 4- to 6-fold lower dose of anti-CTLA-4 improved survival outcomes (45). As a complementary approach to classical antibody-mediated checkpoint blockade, administration of a short-acting peptide (Compound 8) that inhibits PD-1/PD-L1 signaling also provided a significant survival benefit in mice with secondary *C. albicans* sepsis after CLP (31).

The CLP mouse model was further utilized to study the role of PD-1 inhibitors in post-sepsis aspergillosis. While antifungal treatment with amphotericin B alone was not effective to mitigate secondary aspergillosis after CLP, adjunct anti-PD-1 treatment significantly improved 20-day survival and strongly reduced the fungal burden in brain, lung, and kidney tissues (46). However, a limitation of this study was the rather artificial intravenous route of infection with a high inoculum of *A. fumigatus*. Therefore, our group evaluated ICI therapy in neutropenic mice with invasive pulmonary aspergillosis (IPA), the predominant clinical manifestation of invasive aspergillosis in immunocompromised patients (47). Oncological dosing of anti-CTLA-4 or dual checkpoint blockade yielded no significant therapeutic benefit, likely due to exuberant toxicity in a background of acute infection (47). Despite signs of hyperinflammatory toxicity, oncological dosing of anti-PD-1 significantly improved 8-day survival outcomes of mice with IPA. Furthermore, an 8-fold lower dose of anti-PD-1 had considerably attenuated toxicity, provided a significant survival advantage, and improved fungal clearance in mice with IPA compared with isotype- and mock-treated controls (47). Moreover, combination therapy with anti-PD-1 and caspofungin (CAS), a drug that has modest activity against *A. fumigatus* but has been associated with protective immunomodulatory activity (48–50), resulted in a significant additive survival benefit in *A. funigatus*-infected mice compared with either CAS or anti-PD-1 treatment alone (47). Using the same low-dose regimen as in the IPA study, monotherapy with PD-1 pathway inhibitors also improved morbidity and mortality and enhanced fungal clearance in neutropenic mice with invasive pulmonary mucormycosis (IPM) (51). While early protection was comparable between anti-PD-1 and anti-PD-L1, the latter provided more consistent and sustained improvement of morbidity and mortality in mice with IPM (51). Additionally, both genetic PD-1 deficiency and antibody-mediated PD-1 blockade strongly improved survival of mice infected with an otherwise lethal *Histoplasms capsulatum* strain (43).

Altogether, these preclinical studies make a compelling case that ICIs can improve the outcomes of various IFIs in diverse backgrounds of underlying immune dysfunction (i.e., post-sepsis immune exhaustion or pharmacologically induced neutropenia). However, several of these studies also revealed signs of ICI-related immunotoxicities in a setting of acute infection, hyperinflammation, and high antigenic load, especially with high-dose ICIs or dual checkpoint blockade.

### Clinical case reports of ICI therapy in medical mycology

There are very limited clinical data on antifungal ICI therapy, consisting of five case reports:

The first case of adjunct ICI treatment as salvage therapy for an invasive fungal infection was reported in 2017 in a patient with antifungal therapy-refractory mucormycosis (52). A previously healthy 30-year old woman developed gastrosplenic mucormycosis after suffering a polytrauma and second-degree burns, complicated by sepsis and deep bacterial wound infections. Despite initiation of liposomal amphotericin B and posaconazole and extensive surgical revision with gastrectomy and splenectomy, the patient developed surgically intractable Mucoralean lesions of peritoneal and vascular structures. Because of the poor prognosis and the patient’s state of severe immune paralysis with lymphocytopenia, high expression of PD-1 on Th cells, and signs of poor monocytic maturation, combination immunotherapy consisting of five doses of IFN-γ and a single dose of the PD-1 inhibitor nivolumab was initiated. PD-1 expression reverted to the levels of healthy controls within four days of nivolumab administration. In addition, monocyte maturation and lymphocytopenia improved over a course of two weeks. The patient eventually recovered after several weeks (52).

The second case of antifungal ICI therapy (53) was a 51-year-old woman with relapsed acute myeloid leukemia (AML) after allogeneic hematopoietic stem cell transplantation, who developed fever and sinusitis during chemotherapy-induced pancytopenia. The patient was diagnosed with pan-sinusitis caused by *Lichtheimia ramosa* and *A. fumigatus* and developed progressive mucormycosis with orbital invasion despite initiation of antifungal therapy with liposomal amphotericin B and isavuconazole, several surgical interventions, and daily filgrastim (G-CSF). After initiation of nivolumab (four doses in total, every other week) and IFN-γ (ten doses in total, thrice weekly), mucormycosis partially regressed and the patient was discharged. Under nivolumab treatment, markers of lymphocyte activation improved while the expression of PD-1 and CTLA-4 decreased. Although the fungal infection was stable for another month with continued antifungal therapy, the patient developed AML progression and eventually died from septic shock with disseminated intravascular coagulation (53).

In another recent case report (54), combined immunotherapy with nivolumab and IFN-γ was used to treat an extensive polymicrobial soft tissue infection secondary to a minor thoracic trauma in a previously healthy 38-year-old female patient. Pathogens identified by culture and molecular techniques included *Enterococci*, multiple gram-negative bacteria, *Aspergillus* spp., *Mucor* spp., *L. ramosa*, and *Rhizopus arrhizus*. The patient was managed with multiple antibiotics, high-dose liposomal amphotericin B (10 mg/kg/day), triazoles, aggressive surgical debridement, hyperbaric oxygen therapy, and daily IFN-γ injections. Due to organ failures, coagulopathy, persistent detection of *L. ramosa* and *R. arrhizus* in blood and tissue cultures, severe monocyte deactivation, and high T-cellular PD-1 expression, a single dose of nivolumab was given on day 27 after admission. PD-1 expression became negative on the following day, fungal cultures and PCR became negative over the course of one week, and the patient’s clinical status improved rapidly. The patient was discharged after 5.5 months following extensive reconstructive surgery (54).

Similarly, combinatorial immunotherapy with nivolumab and IFN-γ was reported in a 56-year-old diabetic patient with COVID-19 who had received dexamethasone and tocilizumab (anti-IL-6) and developed invasive pulmonary aspergillosis one week after intensive-care unit (ICU) admission, later complicated by two Mucoralean brain abscesses and ethmoidal sinusitis (55). The brain lesions were insufficiently responsive to combined antifungal therapy with liposomal amphotericin B and triazoles, and only partially accessible for surgical debridement. Neutrophil and lymphocyte counts were normal to high, but both Th cells and CTLs displayed strong PD-1 expression. The patient received two doses of nivolumab 4 weeks apart and IFN-γ thrice weekly for four weeks. Subsequently, PD-1 expression decreased, CTL proliferation modestly increased, and cerebral abscesses decreased in size. However, the patient developed ventilation-associated pneumonia with septic shock, oliguric renal failure, and hepatic cytolysis, necessitating discontinuation of liposomal amphotericin B and immunotherapy. Under isavuconazole only, cerebral mucormycosis progressed and the patient deteriorated. The authors discussed that earlier salvage immunotherapy might have had a stronger immunological and clinical impact and underscored the need to study optimal timing of antifungal ICI therapy in the setting of underlying antifungal therapy (55).

Additionally, the first case of nivolumab treatment in a patient with invasive fusariosis was reported recently (56). A 55-year old male AML patient undergoing high-dose chemotherapy developed neutropenic fever and pneumonia and was diagnosed with disseminated fusariosis caused by *Fusarium solani*. The patient received aggressive antifungal therapy with liposomal amphotericin B and voriconazole and was treated on the ICU for several weeks. Eleven weeks later, the patient developed hepatosplenic fungal lesions. As renal toxicities limited amphotericin B dosing, voriconazole remained sub-therapeutic, and the hepatosplenic lesions showed high expression of PD-L1, nivolumab was initiated as salvage treatment (four doses in total, every other week). PD-1 expression on T cells strongly declined after initiation of nivolumab, whereas T-helper-cell concentrations (especially Th1 cells) in peripheral blood increased. Furthermore, signals of enhanced CTL and NK-cell activation were seen. Radiological, histopathological, and microbiological examination confirmed resolution of the fungal lesions and showed no evidence of ongoing fusariosis. Liposomal amphotericin was continued on an outpatient basis for twelve months and no recurrence of fungal infection was seen. However, the patient developed adrenal insufficiency, likely as a side effect of the ICI treatment (56).

While these case reports suggest a role of ICIs as a “last resort” immunotherapeutic intervention, therapeutic success relied on a combination with extensive surgery and/or aggressive conventional antifungal treatment. Additionally, combination immunotherapy with other immunomodulators was used in four out of five case reports. Furthermore, there is a possibility of “publication bias”. Larger case series or interventional trials with more thorough immune phenotyping would be needed to gauge the clinical benefit of ICIs in medical mycology. Notably, the few published cases suggested a trend of more favorable outcomes in *a priori* immunocompetent patients, i.e., those with trauma-related infections. This early observation highlights the importance to study the impact of underlying malignant diseases and other comorbidities on infection-induced immune exhaustion, responses to antifungal ICI therapy, and ICI toxicities (**Table 1**).

**Table 1:** Future research objectives to address the many unanswered questions about antifungal ICI therapy.

Category	Unanswered question or limitation	Future research objectives
Host-related factors	The role of underlying pharmacological immunosuppression (e.g., corticosteroids) is poorly studied.	Compare the efficacy of antifungal ICI therapy in animal models with different natures and intensities of immunosuppression (e.g., neutropenia, corticosteroids, graft-versus-host disease).
	Immune dysfunction caused by underling hematological malignancies has not been considered in the published preclinical studies of antifungal ICI therapy.	Utilize refined infection models in a background of hematological malignancies (e.g., acute myeloid leukemia) to study both the immunotherapeutic potential of ICIs against opportunistic IFIs and the impact of oncological-intent ICIs on natural antifungal immunity.
	The influence of host genetics (e.g., pentraxin 3 mutation) on the efficacy of ICIs against opportunistic fungi is unknown.	Compare the efficacy of ICIs in mouse strains that are genetically deficient for well-described immunogenetic factors linked to invasive fungal diseases.
	The impact of the host microbiome on the efficacy of antifungal ICI therapy has not been studied.	Study antifungal ICI therapy in non-sterile (pet shop) mice with and without antimicrobial pretreatment and perform (intestinal) microbiome profiling; correlate microbiome data and responses to antifungal ICI therapy in patients.
	The influence of underlying comorbidities (e.g., diabetes mellitus) on antifungal ICI therapy remains unknown.	Compare responses to antifungal ICI therapy in mice with representative underlying conditions; thoroughly report underlying conditions in future case series.
Pathogen-related factors	Studies comparing the efficacy of antifungal ICI therapy against pathogens with varying virulence and/or different inoculums are lacking.	Systematically compare the protective activity of ICIs against various clinical and reference isolates and at different inoculums, along with a determination of antigenic burden (e.g., serum galactomannan levels for *Aspergillus*).
	Although common, the impact of co-infections on the efficacy of antifungal ICI therapy and hyperinflammatory toxicities is scarcely studied.	Study antifungal ICI therapy in post-viral IFI models (e.g., post-influenza aspergillosis models) or in models with bacterial & fungal inter-kingdom infections.
	The response of pathogenic fungi to ICI-mediated immune enhancement has not been studied.	Apply dual host & pathogen transcriptomics to ICI- and isotype-treated mice to obtain cues regarding potential fungal defense strategies to the altered immune environment.
Checkpoint targets and ICI agents	The knowledge of the broader landscape of co-stimulatory and co-inhibitory pathways and determinants of immune exhaustion in fungal infections is limited.	Perform thorough and serial immune profiling studies (including “omic” tools) utilizing both animal models and immune cells from patients with IFIs to identify additional checkpoint targets that are upregulated in the response to different pathogenic fungi.
	Studies evaluating additional checkpoint pathways (e.g., TIM-3, LAG-3, TIGIT) or co-stimulatory pathways (e.g., OX40, CD154) as therapeutic targets in medical mycology are lacking.	Conduct proof-of-concept studies for emerging checkpoint targets in representative infection models, with prioritization of targets based on expression kinetics in mice and patients with IFIs.
	Pathways mediating potential resistance to ICI agents are understudied.	Perform serial immune phenotyping after antifungal ICI therapy in animal models or future clinical cases in order to determine compensatory induction of untargeted checkpoint pathways, along with an experimental characterization of their role in antifungal immunity.
Clinical implementation	Optimal timing and sequencing of antifungal ICI therapy has not been studied.	Compare early ICI therapy during the acute infection stage (combined with antifungals) versus ICI therapy after initial treatment with conventional antifungals to reduce the fungal burden and antigenic load.
	ICI dosing is poorly studied and difficult to translate from mice to humans; studies comparing single- and multi-dose regimens are lacking.	Thoroughly evaluate dose-responsiveness in animal models, including humanized dosing schemes, along with a detailed characterization of dose-dependent organ toxicities.
	Specific mechanistic synergies of ICIs with different classes of antifungals remain to be characterized.	Study combinations of ICIs with first-line antifungals and with new investigational antifungal agents.
	ICIs were combined with Th1-polarizing cytokines and hematopoietic growth factors in two case reports, but the merit and mechanistic synergies of such combinations have not been studied in animal models.	Systematically dissect the individual contributions of ICIs and additional immunomodulators to antifungal immunity as well as their potential mechanistic synergies in representative animal models.
Management of toxicities	Off-target toxicities (e.g., colitis, myocarditis, gastritis) of antifungal ICI therapy have not been systematically studied in preclinical models or patients.	Incorporate histopathological assessment and immunohistochemistry of representative organs associated with ICI toxicities in preclinical studies of antifungal ICI therapy
	There are no validated biomarkers to reliably predict antifungal immune augmentation versus risk for toxicities.	Consider detailed immune phenotyping of peripherally assayable materials (e.g., whole blood) as part of preclinical studies and correlate immune features with surrogates of clinical responses (e.g., morbidity scores, fungal burden) and signs of off-target toxicities (e.g., intestinal inflammation).
	Long-term effects of antifungal ICI therapy (e.g., hypersensitivity syndromes, autoimmunity) have not been studied.	As such effects are difficult to study in the currently used preclinical models, longer follow-up reporting (if feasible) would be desirable as part of future clinical case reports or case series.
	The impact of non-corticosteroid agents given to mitigate ICI toxicities (e.g., IL-6 inhibitors or TNF-α inhibitors) on antifungal immune recovery is incompletely understood.	Test combinations (simultaneous and sequential) of ICIs with toxicity-suppressing agents in representative animal models.

### Current mechanistic insights into the protective effects of immune checkpoint blockade during fungal infections and potential immunotoxicities

Mechanistic studies of ICI-driven immune enhancement in fungal infection models mostly focused on exhaustion markers and cytokine responses. Although CTLA-4 blockade inhibited T-cell exhaustion and apoptosis in a murine model of *Candida* sepsis after CLP, no significant impact of ICI therapy on the cytokine environment was found (45). In contrast, others showed increased IFN-γ production by stimulated splenocytes from ICI-treated mice with primary or secondary *Candida albicans* sepsis (32). Interestingly, the authors found concomitant induction of the immunosuppressive Treg cytokine IL-10, especially in anti-PD-1-treated animals, along with strong elevations of the proinflammatory cytokine IL-6 (32), which has been linked to both protective anti-yeast responses (57) and immunotherapy-related immunotoxicities (58).

The dualism of presumably protective and adverse cytokine responses after ICI therapy was confirmed in mice with IPM, where blockade of the PD-1/PD-L1 pathway led to modest elevations of proinflammatory cytokines and chemokines associated with fungal clearance (e.g., GM-CSF, TNF-α), paralleled by signals of enhanced Th2 (IL-5, IL-13) and Treg (IL-10) cytokine responses after ICI treatment (51). Additionally, anti-PD-1-treated mice with IPM had massively elevated serum levels of IL-6 and other proinflammatory cytokines that could contribute to both protective immune cell recruitment and immunotoxicities (51).

Multiplex cytokine profiling in lung homogenates from anti-PD-1-treated mice with IPA revealed significant induction of key pro-inflammatory cytokines (e.g., TNF-α, IL-1β) and neutrophil-attracting chemokines (e.g., CXCL2) compared with isotype-treated controls (47). Consistent with these findings, hyphal invasion foci in anti-PD-1-treated mice with IPA were surrounded by more dense leukocyte infiltrates, which displayed an increased neutrophil-to-lymphoid cell ratio compared with isotype-treated infected mice (47).

Immune phenotyping in anti-PD-1-treated mice with PJP revealed heterogenous results. While global immune cell infiltration into the lung tissue increased after anti-PD-1 treatment, the percentage of neutrophils decreased in both the lung and bloodstream (36). However, these measurements were performed 3 weeks post-infection and might not have captured the full picture of immune cell kinetics, especially the early trends (59).

Few studies provided additional non-cytokine surrogates of APC activation. Both anti-PD-1 and anti-PD-L1 enhanced the fungal killing potential of *ex-vivo* splenocytes from mice with IPM compared with isotype treatments (51). APCs from anti-PD-1-treated mice with post-sepsis aspergillosis had significantly restored expression of the maturation marker CD83 compared with mock-treated infected controls, resembling expression levels in uninfected animals (46). Similarly, DCs and macrophages of anti-PD-L1-treated mice with secondary *C. albicans* sepsis showed increased expression of major histocompatibility (MHC) II molecules, which are pivotal for antigen presentation to Th cells (32). In both studies, ICI-mediated enhancement of APC activation and maturation was paralleled by reinvigoration of IFN-γ release from re-stimulated *ex-vivo* T cells, further supporting that ICIs can restore exhausted APC/T-cell feedback loops during fungal infections (32, 46). In contrast, anti-PD-1 did not alter the pulmonary accumulation or activation profile of myeloid cells in mice with persistent cryptococcal lung infection, but enhanced activation of Th1 and Th17 cells while dampening the production of Th2 and Treg cytokines (35). Similarly, genetically PD-1-deficient mice had significantly enhanced Th1 and Th17 responses during *Pneumocystis* infection, paralleled by signals of macrophage polarization toward a protective M1 phenotype and strong upregulation of both pro- and anti-inflammatory cytokines (36). However, these readouts were not studied after pharmacological PD-1 blockade (36).

Although some surrogates of adverse immune alterations (e.g., high serum levels of IL-6) have been described, the determinants of immunotoxicities after antifungal ICI therapy are largely unknown. The immunopathogenesis of IFIs often comprises a complex combination of immunosuppression and exhaustion with topical and systemic inflammation. Patients whose pathogenesis is predominantly driven by hyperinflammation rather than immune exhaustion might not benefit from ICIs but potentially experience worsening of immunopathology and progressive infection. For instance, a case of acute exacerbation and progression of chronic pulmonary aspergillosis has been described in a patient receiving nivolumab to treat lung cancer (60).

As a severe form, hyperinflammatory toxicities can manifest as an immune reconstitution inflammatory syndrome (IRIS), a clinical entity observed in immunocompromised patients who experience sudden restoration of immune activity, e.g., due to abrupt tapering of corticosteroids or initiation of highly active antiretroviral therapy in patients with HIV infection (61). IRIS has been described after ICI use in patients with underlying opportunistic infections (e.g., tuberculosis) and can result in severe systemic inflammation, overshooting immune responses to the underlying infection, and tissue damage (19, 62).

Additionally, cancer immunology studies have described a multitude of autoimmune toxicities of ICIs that can affect essentially any organ, as extensively reviewed elsewhere (63). While colitis, diarrhea, dermatological events, and endocrine toxicities are the commonest irAEs in patients receiving ICIs as cancer immunotherapy (63), manifestations of autoimmune toxicities after anti-infectious ICI therapy remain to be characterized. Notably, management of autoimmune toxicities after ICI therapy often requires corticosteroids and/or other immune-attenuating agents (e.g., TNF-α inhibitors) that may in turn aggravate an underlying IFI (19).

**Figure 2:**
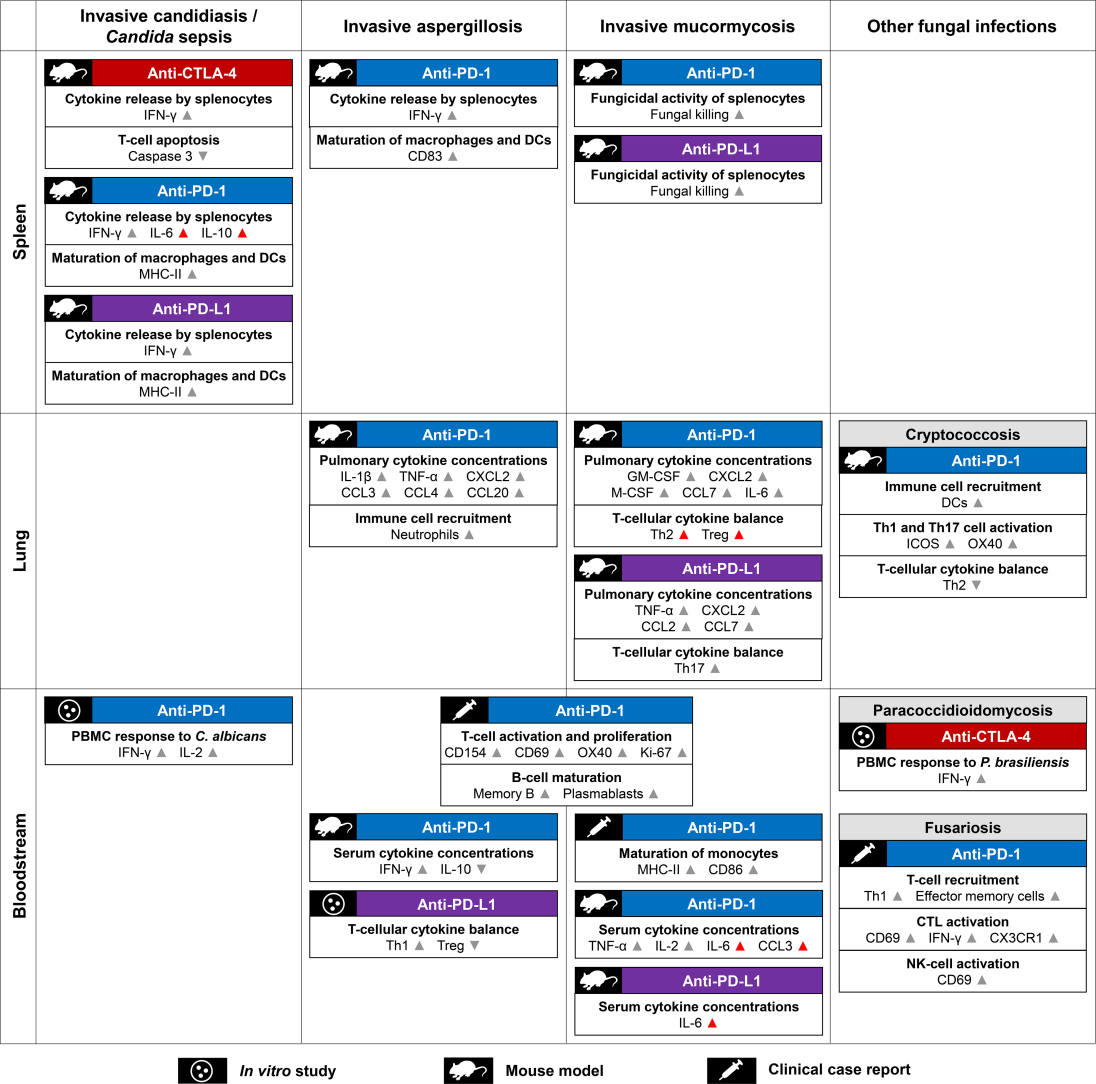
Summary of proposed mechanisms contributing to checkpoint inhibitor-mediated immune enhancement against opportunistic fungal pathogens. Mechanistic readouts are grouped by type of infection and the body site of specimen collection. Grey arrowheads indicate changes to key readouts likely associated with antifungal immune enhancement. Unfavorable responses (e.g., Th2 and Treg activation) and immunological changes potentially linked to toxicities (e.g., high concentrations of serum IL-6) are indicated by red arrowheads. Abbreviations: CD, cluster of differentiation; CTL, cytotoxic T lymphocyte; CTLA-4, Cytotoxic T-Lymphocyte Antigen 4; C(X)CL, C-(X-)C-motif chemokine receptor ligand; CX3CR1, C-X3-C-motif Chemokine Receptor 1; DC, dendritic cell; (G)M-CSF, (Granulocyte) Macrophage Colony Stimulating Factor; IFN, interferon; IL, interleukin; MHC, Major Histocompatibility Complex; NK cell, natural killer cell; PBMCs, peripheral blood mononuclear cells; PD-1, Programmed Cell Death Protein 1; PD-L1, Programmed Death Ligand 1; Th(1/2/17), (type 1/2/17) T-helper cells; Treg, regulatory T cells.

In summary, while toxicities are poorly understood, limited data from preclinical models and patients with IFIs suggest that the immune environment created by ICI treatment could promote clearance of opportunistic fungi through a combination of increased innate immune cell recruitment, restored APC/T-cell interactions, and enhanced maturation and fungicidal activity of APCs (**Figure 2**). However, the detailed underpinnings of ICI-mediated changes to the immune environment at the site of infection are still poorly understood, as are the long-term effects of antifungal ICI therapy and the determinants of immunotoxicities. These gaps of knowledge hamper the identification of much-needed (immune) biomarkers to identify patient cohorts who would benefit most from antifungal ICI therapy.

## Research gaps and future directions

### Do the current animal models paint an accurate picture of the merits of ICIs in medical mycology?

Pharmacologically immunosuppressed but otherwise healthy rodent models are widely used for preclinical fungal immunology studies (64, 65), including several studies on ICI therapy discussed above. While being relatively simple, cost-efficient, and generally well reproducible, these models often fail to adequately reflect the underlying immune dysfunction of high-risk patients, e.g., those with acute leukemia. Consequently, results cannot be extrapolated to other host environments without additional experimental validation (64, 65), creating a major unmet need to study the impact of ICIs on the outcomes and immunopathology of opportunistic mycoses in pathophysiologically relevant preclinical models, e.g., leukemic mice (66).

Although most models of *Candida* sepsis do not rely on pharmacological immunosuppression, there are three common limitations. On the one hand, preclinical sepsis models often utilize intravenous inocula of millions or billions of yeast cells per kg body weight, especially when assessing pathogens with relatively low virulence such as *Candida auris* (34, 67). Such unnaturally high inocula can result in overwhelming infection (68) and are a considerable confounder of immunological responses and exhaustion phenotypes compared to real-life clinical scenarios. Additionally, the published studies on ICI therapy in single-hit or CLP *Candida* sepsis models utilized *a priori* healthy mice. As particularly high mortality of *Candida* sepsis has been reported in immunocompromised patients and in patients with prior antibiotic treatment, co-infections, obesity, preexisting cardiovascular diseases, diabetes mellitus, or major surgery (69, 70), recapitulation of these conditions in more nuanced mammalian models would be needed. Lastly, fungal sepsis is frequently associated with indwelling catheters, resulting in immune-resistant yeast biofilms (71). As such biofilms stimulate inflammatory host responses while immunological clearance is inefficient, catheter-associated infections could be particularly prone to immune exhaustion (71). However, studies supporting that ICIs can promote a meaningful immune response against yeasts engulfed in catheter-associated biofilms are lacking.

Fungal infection models are often designed to produce an acutely lethal infection (68), which might underestimate the role of protracted immune exhaustion while exaggerating hyperinflammatory toxicities during the initial inflammatory phase of infection. Thus, it would be desirable to validate the efficacy of ICIs in preclinical models across a broader range of inocula and by using different fungal isolates with varying virulence. Another major limitation of all published animal studies is the initiation of antifungal ICI therapy within few hours to days after fungal infection, aiming to intercept immune exhaustion rather than attempting to revert an exhausted immune landscape. Although yielding promising results, this approach is rather artificial in view of the often difficult and delayed diagnosis of IFIs. Additionally, early ICI therapy in a background of high fungal burden and inflammation likely contributed to the reports of considerable toxicities, especially at full oncological dosing of ICIs (45, 47). In contrast, all clinical case reports described late-stage application of ICIs in patients with IMIs, a strategy that might have suboptimal efficacy in a setting of severely exhausted and terminally differentiated effector cells (55). Therefore, the optimal timing of ICI therapy needs to be thoroughly studied in preclinical models. Other common limitations of most published preclinical studies on antifungal ICI therapy included the use of a single dosing regimen (19), the omission of first-line antifungal therapy as the “standard of care”, the utilization of inbred mice, and performance of studies in semi-sterile facilities without natural mold exposure (72, 73). The latter two limitations bear additional significance in view of the increasingly recognized modulation of treatment responses to oncological ICI therapy by variations in the host microbiome (74, 75).

Despite these limitations, there has been considerable agreement among the published studies in transgenic (e.g., PD-1-deficient) mice, immunotherapeutic studies in murine infection models, assessment of *ex-vivo* human and murine immune cells, and clinical case reports regarding the effects of checkpoint induction and inhibition in a broad spectrum of IFIs. Nonetheless, more refined models with increasing incorporation of underlying conditions such as leukemia (66) or co-infections (76) would not only provide a more representative host environment but could also play a major role in defining specific host populations that might particularly benefit from antifungal ICI therapy.

### Are PD-1, PD-L1, and CTLA-4 the only checkpoint targets for antifungal ICI therapy?

Beyond the PD-1/PD-L1 and CTLA-4 pathways, an increasing number of preclinical and clinical immunooncology studies explore the merits of additional checkpoint targets to mount broad anti-tumor immunity and mitigate treatment resistance. Co-inhibitory pathways can be subdivided depending on their involvement in i) interactions of APCs and Th cells, ii) interactions of APCs and CTLs, and iii) interactions of CTLs and somatic, non-lymphoid cells, such as tumor cells or infected tissue (77, 78). We would hypothesize that molecules involved in the Th/APC interplay (**Figure 1B**) are the most promising targets to treat IFIs, since the role of CTLs in natural antifungal immunity is less clearly defined than the multifaceted Th responses (39, 79). Notably, checkpoint pathways with a known role in the APC/Th interplay, such as TIM-3, LAG-3, and TIGIT, are among the most promising checkpoint targets in the cancer immunotherapy pipeline (80). Furthermore, there is some evidence for a role of these targets in the host response to (viral) infections, including potential synergies with conventional checkpoint targets such as PD-1 (81, 82). Therefore, detailed exploration of these targets in preclinical models of fungal infections and in *ex-vivo* samples from infected patients would be warranted.

Beyond their role as independent therapeutic targets, other checkpoint pathways were shown to drive resistance to PD-1- or CTLA-4-targeted oncological ICI therapy (83). Data about resistance formation to antifungal ICI therapy are scarce, as most of the published preclinical studies used relatively short follow-up periods and detailed information on checkpoint marker expression beyond PD-1/PD-L1 was provided only in a single clinical case report (53). Nonetheless, there are signals suggesting potential paths to ICI resistance in fungal infections. For instance, immune phenotyping of *ex-vivo* T cells from patients with IC suggested co-induction of multiple checkpoint pathways, posing potential limitations to single-agent ICI therapy (29). PD-1/PD-L1 pathway blockade in a murine mucormycosis model successfully suppressed the targeted molecules and co-suppressed CTLA-4 but led to rapid upregulation of TIM-3 within 4 days of ICI initiation (51). Likewise, nivolumab therapy in a patient with aspergillosis and mucormycosis caused an initial upregulation of other checkpoint pathways, such as TIM-3 and LAG-3 (53). Both pathways are well-known drivers of resistance to oncological ICI therapy (84). These findings deserve further study in preclinical infection models in order to better understand the kinetics and implications of compensatory checkpoint induction after antifungal ICI therapy.

As an alternative strategy to releasing the “immunological brake” through ICIs, agonists of co-stimulatory molecules such as CD28, CD134/OX40, CD137, or CD154/CD40L have been investigated in preclinical oncology studies and are currently undergoing early-phase clinical evaluation (85, 86). These molecules have pleiotropic effects on early immune activation and can potentially synergize with ICIs (85, 86). Of note, most of these targets have well-defined functions in the immune response to pathogenic fungi. For instance, CD154 is crucial for the initiation and enhancement of antifungal host defense and has been studied as a target for investigational immune diagnostics in medical mycology (87, 88). Additionally, both CD137 and CD154 have been previously proposed as reliable selection markers for the enrichment of mold-reactive T-cells for adoptive transfer (8, 89). It is therefore conceivable that co-stimulation-enhancing agents could confer increased protection against fungal pathogens. However, these agents are associated with significant toxicities and are prone to causing cytokine release syndrome (85, 86). Although new fusion proteins or topical application could reduce these side effects (85), the narrow therapeutic index of co-stimulation-enhancing agents is concerning for their use in an acute infection setting. Nonetheless, as these agents are increasingly introduced into the oncological treatment landscape, it will be important to investigate their effects on host defense against opportunistic pathogens and their potential for immunotherapeutic applications in medical mycology.

### Are there benefits of ICI-based combination immunotherapy?

As discussed above, dosing of ICIs during acute infection and inflammation is limited by toxicity concerns and immunomodulators are unlikely to be applied without concomitant conventional antifungal therapy. All three major classes of modern antifungals – lipid amphotericin B, azoles, and echinocandins – have known immunopharmacological effects that could be implicated in potential synergies with ICIs. These immunomodulatory effects are best-characterized for echinocandins (48, 49). Echinocandins disturb the equilibrium of the fungal cell wall and expose beta-glucans that are considered the most potent fungal stimulus of innate immune cell activation (48, 49, 90). Echinocandins can thereby enhance fungicidal activity of mononuclear phagocytes and neutrophils and induce proinflammatory cytokine secretion (48–50, 91). We have previously described a significant additive survival advantage of PD-1 blockade and CAS in our murine IPA model despite the poor monotherapeutic activity of CAS against *A. fumigatus* (47). Several promising investigational antifungal drugs, especially rezafungin and ibrexafungerp, have known cell well-altering properties (3) and are therefore likely to exert similar immunomodulatory effects that might provide synergies with ICIs. Triazoles can induce upregulation of the pattern recognition receptors TLR2 and TLR4 (48) that are pivotal for activation of neutrophils and mononuclear phagocytes by fungal antigens (79). Although mechanistic synergies with ICIs remain to be defined, anti-PD-1 provided an added benefit when combined with fluconazole in a *C. albicans* sepsis model (32). Immunomodulatory properties of lipid amphotericin B formulations are predominantly associated with the liposomal packaging (92, 93). Liposomes can stimulate APCs, modulate TLR expression, and promote nonoxidative fungal killing by neutrophils (92, 93). Consequently, pretreatment with empty liposomes improved survival and attenuated immune injury in a murine IPA model (92). Given the presumed effects of ICIs on neutrophil recruitment and enhancement of phagocytic activity of APCs, immunological synergism with liposomal amphotericin B is conceivable and deserves further study.

Four clinical reports of antifungal ICI therapy further suggested that combinatorial immunomodulation strategies, especially the combination of ICIs and the Th1-polarizing cytokine IFN-γ, could provide additional benefits (52–55). IFN-γ has been previously used as salvage therapy in patients with fungal infections in a background of dysfunctional immunity (13, 94). For instance, IFN-γ was well-tolerated in patients with *C. albicans* sepsis and partially restored MHC II expression and secretion of TNF-α and IL-1β (13). Oncological studies are exploring synergies between ICIs and IFN-γ in preclinical models and in patients with advanced solid tumors (95). Notably, ICIs alone did not enhance Th1 cytokine release in our IPA and IPM models (47, 51), supporting a potential independent benefit of IFN-γ in ICI-based combination immunotherapy. Additionally, hematopoietic growth factors such as G-CSF and GM-CSF (6, 11, 14–18), whose endogenous levels in mice were only modestly induced by antifungal ICI therapy alone (47, 51), are potentially interesting targets for antifungal combination immunotherapy. However, thorough preclinical studies are needed to determine the risk of additive immunotoxicities when combining ICIs with additional immunomodulators.

Additionally, combinations of ICIs with adoptive T-cell transfer and chimeric antigen receptor T cells have been studied as investigational oncological salvage treatments (96). *Ex-vivo* expansion of cellular products, as proposed for immunotherapeutic applications in medical mycology (9), often requires prolonged antigen challenge and/or the use of stimulatory cytokine cocktails. These manufacturing-related factors and subsequent encounters with a suppressive *in-vivo* environment can promote exhaustion of the infused cells (97, 98). Although PD-1 pathway blockade was shown to augment the efficacy of CAR T-cell therapy against various malignancies (99, 100), CAR T-cell exhaustion might eventually be overcome by engineering of exhaustion-resistant cells (98). In contrast, concomitant checkpoint blockade could have merits for adoptive transfer of unmodified, enriched, allogenic antigen-reactive T cells (8, 89), a methodology developed to clinical scale and tested in small, early-phase studies for mycological applications (101, 102).

The strategies discussed above predominantly focus on strengthening ICI-mediated immune augmentation through synergistic immune interventions. Additionally, oncological trials are increasingly exploring combinatorial treatment strategies that aim to decouple protective anti-tumor responses from detrimental off-target toxicities. IL-6 emerged as an interesting target in this context (103). Given that studies of ICI therapy in preclinical models of fungal infections showed strong induction of IL-6 (32, 51), such approaches warrant further study in medical mycology. On the other hand, IL-6 inhibitors are associated with an increased fungal infection risk, especially in COVID-19 patients (55, 104). Therefore, dosing and timing relative to the ICI treatment will likely determine the impact of IL-6 inhibitors on the delicate balance between fungal clearance, suppression of antifungal immunity, and the level of immunotoxicities. A similar dualism could be seen with inhibitors of CXCR1 activation by CCL3. This chemokine showed strongly elevated serum levels in mice with IPM receiving anti-PD-1 and could be implicated in immunotoxicities (51). Prior research by our group showed improved survival and attenuation of hyperinflammatory tissue injury in mice with disseminated candidiasis receiving a CXCR1/CCL3 inhibitor during the initial inflammatory stage (105); however, combined application with ICIs during fungal infections remains to be studied.

### Can we translate hallmarks of ICI-induced changes in the tumor microenvironment to infected tissue?

Both the general rationale for a merit of ICIs in medical mycology and potential future strategies for combination immunotherapy adapted from oncological research partially rely on commonalities between the immune environment of infected tissue and cancers (106). As extensively reviewed elsewhere, these commonalities include similar triggers (e.g., nucleic acids and other damage-induced molecular patterns), receptor signaling pathways (e.g., a major role of TLR signaling), and features of immune exhaustion (19, 106).

Some features of the tumor microenvironment that are hallmarks of protective responses to immunotherapy (77, 107) are also known to be crucial for host defense against fungal pathogens. For example, DC and macrophage polarization can shape the ICI-induced antitumor response (107). Cancer immunology studies suggested that M1 macrophages favor antitumor immunity through NK-cell and CTL activation and are associated with therapeutic response to ICIs, whereas most subsets of M2 macrophages elicit pro-angiogenetic and immunosuppressive effects (107–109). Similarly, M1-polarization is considered beneficial in antifungal immunity and has been linked to increased NK-cell activation, phagocytic capacity, and secretion of IFN-γ in the response to *A. fumigatus* (110) and *C. albicans* (111).

On the other hand, we need to be aware of major differences between the tumor and infection environment. Clearance of tumor cells strongly relies on CTL effector responses and the density of functional CTLs is a major predictor of ICI efficacy against various tumors (112, 113). In contrast, little is known about the contributions of CTLs during antifungal ICI therapy. Additionally, there are major differences between the myeloid cell repertoire associated with fungal elimination (e.g., polymorphonuclear neutrophils or alveolar macrophages) and the mostly immunosuppressive myeloid-derived cell populations dominating the tumor environment (106).

Another distinct difference between host immunity to many fungal pathogens and cancers is the constant exposure to fungal commensals and aero-antigens. For instance, chronic occupational mold exposure has been shown to modulate the T-cell repertoire and cytokine responses to *Aspergillus* antigens (114, 115). Similarly, fungal commensals, especially in the intestinal microbiome, are powerful modulators of the antifungal T-cell repertoire (116, 117). It remains unclear and difficult to study in the currently used animal models how prior encounters with fungal antigens and the mycobiome of fungal commensals impact responses to antifungal ICI therapy.

Studies describing potential mechanisms of ICI-mediated antifungal immune enhancement (section 2.4) were restricted to analyses of host biomarkers and did not assess changes in fungal responses to the treatment. However, immune-oncologists described the ICI-enhanced immune environment as a Darwinian scenario for tumor cells, which are often not entirely eradicated and either become more aggressive or deploy immunoinhibitory mechanisms to undermine host defense (106). Tumor cells possess a panoply of strategies to suppress inflammatory responses and evade ICI-augmented host immunity, e.g., through suppression of antigen expression or modulation of the cytokine environment (106). Although similar scenarios would be conceivable during fungal infection in view of the many known immune-evasive mechanisms of opportunistic fungi (118, 119), specific changes in fungal metabolism during ICI-induced immune stress remain to be identified. Such effects might also be triggered by biochemical features of the surrounding tissue. For instance, hypoxia is a known contributor to oncological ICI resistance (120) and has pleiotropic effects on host-pathogen interplay during IFIs (121), including potential attenuation of antifungal DC responses (122), fungal cell wall alterations associated with immune evasion (123), and homeostatic changes in angiogenesis (124). As these processes are highly dynamic, serial application of increasingly powerful “omic” technologies including dual host-pathogen transcriptomics and spatial proteomics should be employed to shed light into both the versatile host immune environment and fungal reprogramming during ICI therapy.

### How does oncological ICI therapy impact natural immunity to opportunistic fungi in high-risk patients?

This review focused on therapeutic-intent checkpoint blockade after a fungal infection has been contracted or even as a last resort after unsuccessful conventional antifungal therapy. In contrast, the impact of oncological ICI therapy on the epidemiology and immunopathology of IFIs is largely unexplored. After their tremendous success in the treatment of various solid tumors, ICIs are now increasingly investigated as an immunotherapeutic option in patients with hematological malignancies who are at the highest risk for fungal infections, especially patients with AML (125–127). Although the mono-therapeutic activity of ICIs against AML is relatively poor (126), ICIs might have a promising role as part of combination therapy with hypomethylating agents, especially in elderly patients or those with co-morbidities who cannot tolerate high-intensity cytotoxic chemotherapy (125–127). For instance, a single-arm phase 2 trial of 5-azacytidine (5-AC) plus nivolumab showed encouraging response rates and survival outcomes in patients with relapsed/refractory AML and randomized phase 3 trials are in progress (126, 128).

We have previously proposed the “double-hit hypothesis” that ICIs given as part of AML remission-induction therapy might provide an additional “off-target” benefit by improving the outcomes of opportunistic fungal infections in leukemic patients (125). Specifically, anti-leukemia ICI therapy could reduce the risk and severity of opportunistic infections through facilitation of less-cytotoxic conventional chemotherapy (125), improved remission rates (126, 127), and direct enhancement of antifungal immune responses (see section 2.4). However, this hypothesis has not been studied in clinical trials or animal models. Therefore, there is a need to employ preclinical leukemia models (see section 3.1) to study the effects of different chemotherapy regimens with and without ICIs on the outcomes of opportunistic mold infections during remission-induction chemotherapy (RIC). Furthermore, recently developed, holistic (whole blood-based) *ex-vivo* assays of antigen-specific immunity (129, 130) should be employed to compare innate and adaptive immunity to pathogenic fungi in preclinical models and leukemia patients receiving RIC with and without concomitant ICIs. Given the rapid emergence of new targeted anti-leukemia therapies, such studies should be further expanded to CD47 macrophage checkpoint inhibitors (131) and the growing number of anti-leukemic small molecules with potential implications for antifungal immunity (132).

## Conclusions

A growing body of studies has documented a role of immune exhaustion and the induction of immune checkpoint pathways during opportunistic infections due to various classes of pathogenic fungi. In addition, several preclinical studies suggested protective therapeutic activity of classical immune checkpoint inhibitors (i.e., PD-1, PD-L1, and/or CTLA-4 inhibitors) in murine models of fungal sepsis, invasive mold infections, or dimorphic fungal infections. Positive effects of antifungal ICI therapy on innate immune cell recruitment, activation and maturation of innate immune cells, T-cell activation and exhaustion, and cytokine production have been described. Although some commonalities between the tumor and infection environment have been established, many unknowns remain regarding global, pathogen-specific, and organ-specific changes to the infection microenvironment during antifungal ICI therapy as well as the fungal response to ICI-induced immune enhancement (**Table 1**). Furthermore, the narrow therapeutic index and risk of detrimental immunotoxicities remain major challenges for broader clinical translation of antifungal ICI therapy. Systematic preclinical studies of timing, dosing, synergies with first-line antifungals and other immunomodulators, and evaluation of emerging ICIs in more nuanced animal models are needed to expand our data framework to inform future clinical applications of antifungal ICI therapy (**Table 1**). Furthermore, the development of host biomarkers will be pivotal to guide patient stratification and predict successful augmentation of antifungal immunity and potential immunotoxicities. Beyond further studies of the immunotherapeutic merits and implementation of ICIs in opportunistic fungal infections, we need to expand our understanding of the effect of ICIs given as part of oncological (e.g., anti-leukemia) therapies on antifungal immunity and the course of opportunistic IFIs in high-risk patients.

## Author contributions

SW and DK reviewed the fungal immunology literature, wrote and edited the paper. SSW provided additional input regarding immuno-oncological studies and their potential implications for antifungal ICI therapy. All authors contributed to the article and approved the submitted version.

## Funding

This manuscript was supported by the MD Anderson Cancer Center, Division of Internal Medicine Research and Quality Improvement Award (to SW), an MD Anderson Cancer Center Institutional Research Grant (award number 2022-00060729-Y1 to SW), NIH funding (R01 AI133822 and R56 AI109294 to SSW; R03 AI166285 to DK), and the Robert C. Hickey Chair for Clinical Care endowment (to DK).

## Acknowledgments

We thank Jordan Pietz (MD Anderson Cancer Center, Creative Communications) for input regarding data visualization.

## Conflict of interest

SSW reports consultant fees from Asylia Therapeutics Inc and Cellino Biotech Inc. DK reports honoraria and research support from Gilead Sciences and Astellas Pharma. He received consultant fees from Astellas Pharma, Merck, and Gilead Sciences, and is a member of the Data Review Committee of Cidara Therapeutics, AbbVie, Scynexis, and the Mycoses Study Group.

The remaining author declares that the research was conducted in the absence of any commercial or financial relationships that could be construed as a potential conflict of interest.

## Publisher’s note

All claims expressed in this article are solely those of the authors and do not necessarily represent those of their affiliated organizations, or those of the publisher, the editors and the reviewers. Any product that may be evaluated in this article, or claim that may be made by its manufacturer, is not guaranteed or endorsed by the publisher.
